# Associations of race and ethnicity with risk of developing invasive breast cancer after lobular carcinoma in situ

**DOI:** 10.1186/s13058-019-1219-8

**Published:** 2019-11-14

**Authors:** Vanessa Dania, Ying Liu, Foluso Ademuyiwa, Jason D. Weber, Graham A. Colditz

**Affiliations:** 1 Division of Public Health Sciences, Department of Surgery, Washington University School of Medicine, 660 South Euclid Avenue, Campus Box 8100, St. Louis, MO 63110 USA; 2 Alvin J. Siteman Cancer Center at Barnes-Jewish Hospital and Washington University School of Medicine, St. Louis, MO USA; 3 Oncology Division, Department of Medicine, Washington University School of Medicine, St. Louis, MO USA; 4 Division of Molecular Oncology, Department of Medicine, Washington University School of Medicine, St. Louis, MO USA

**Keywords:** Breast cancer, Lobular carcinoma in situ, SEER, Race, Second primary cancer

## Abstract

**Background:**

Lobular carcinoma in situ (LCIS) of the breast is a risk factor of developing invasive breast cancer. We evaluated the racial differences in the risks of subsequent invasive breast cancer following LCIS.

**Methods:**

We utilized data from the Surveillance, Epidemiology, and End Results registries to identify 18,835 women diagnosed with LCIS from 1990 to 2015. Cox proportional hazards regression was used to estimate race/ethnicity-associated hazard ratios (HRs) and corresponding 95% confidence intervals (CIs) of subsequent invasive breast cancer.

**Results:**

During a median follow-up of 90 months, 1567 patients developed invasive breast cancer. The 10-year incidence was 7.9% for Asians, 8.2% for Hispanics, 9.3% for whites, and 11.2% for blacks (*P* = 0.046). Compared to white women, black women had significantly elevated risks of subsequent invasive breast cancer (HR 1.33; 95% CI 1.11, 1.59), and invasive cancer in the ipsilateral breast (HR 1.37; 95% CI 1.08, 1.72) and in the contralateral breast (HR 1.33; 95% CI 1.00, 1.76). Black women had significantly higher risks of invasive subtypes negative for both estrogen receptor and progesterone receptor (HR 1.86; 95% CI 1.14, 3.03) and invasive subtypes positive for one or both of receptors (HR 1.30; 95% CI 1.07, 1.59). The risk of subsequent invasive breast cancer was comparable in Asian women and Hispanic women compared with white women.

**Conclusions:**

Black women had a significantly higher risk of developing invasive breast cancer, including both hormone receptor-positive and hormone receptor-negative subtypes, after LCIS compared with white counterparts. It provides an opportunity to address health disparities.

## Background

Lobular carcinoma in situ (LCIS) is a pre-malignant breast lesion that increases a woman’s long-term risk of developing invasive carcinoma [1–5]. Compared to the general population, women diagnosed with LCIS have a 3-fold to 10-fold higher risk of invasive breast carcinoma [1–7]. The annual incidence of subsequent breast cancer in women diagnosed with LCIS is 1 to 2% per year [6, 8]. LCIS is largely diagnosed in women between the ages of 45 to 50 years old [8, 9]. LCIS does not commonly present with palpable breast masses or calcification on mammography; only 0.5 to 3.8% of benign breast biopsies are diagnosed with LCIS [7]. Prior analysis of National Cancer Institute’s Surveillance, Epidemiology, and End Results (SEER) data has shown that the age-adjusted incidence of LCIS increased from 2.0 per 100,000 in 2000 to 2.75 per 100,000 in 2009 [10]. This may be due to increased surveillance and utilization of mammography.

Although considered as low-risk pre-invasive or benign lesions, controversy persists regarding whether to classify LCIS as a precursor of subsequent invasive carcinoma or just as a lesion that increases the risk of breast cancer in a woman’s lifetime [1, 9, 11]. This abnormal proliferation of cells frequently originates in the terminal ductal lobular unit of the breast and may spread to the ducts [12]. LCIS may also be associated with the development of invasive ductal carcinoma, typically estrogen receptor-positive (ER+) lesions [13].

Pathological models of breast cancer progression have proposed distinct ductal and lobular pathways from normal breast tissue to invasive breast cancer [14]. Ductal carcinoma in situ (DCIS) and LCIS are pre-invasive breast lesions among the two proposed pathways to invasive breast cancer. We and other groups have previously shown that among African American women, DCIS is associated with excess risk of subsequent invasive breast cancer when compared to white women [15–20]. Potential racial differences in the risk of subsequent invasive breast cancer following LCIS are far less understood. In this study, we examined the associations between race/ethnicity and the risks of subsequent invasive breast cancer in a population-based racially diverse group of women with LCIS. Understanding of racial differences in LCIS outcomes will contribute to the development of patient management strategies and refine our understanding of disparities in breast cancer incidence/risk.

## Methods

### Patient selection

The cases included in this study were from the SEER database. The data were derived from 17 SEER registries which represent approximately 28% of the US population. De-identified SEER data were utilized thus exempting the study from review by our Institutional Review Board. The analysis included women diagnosed with primary unilateral LCIS (no concurrent DCIS) between January 1990 and June 2015 who had no prior cancer history, were aged 20 or older, and were followed for at least 6 months (*n* = 20,021). Patients who underwent bilateral mastectomy were excluded (*n* = 716). Race/ethnicity was determined as mutually exclusive categories of non-Hispanic white (hereafter acknowledged as white), non-Hispanic black (black), non-Hispanic Asian (Asian), and Hispanic. The analysis excluded Pacific Islanders and other racial classifications (*n* = 470). Thus, 18,835 women with LCIS were included in the analysis.

### Outcomes

The primary outcome of interest is subsequent invasive breast cancer, defined as invasive breast cancer, regardless of histological features, in either of the breasts or metastatic breast cancer that was diagnosed at least 6 months after the initial LCIS to ensure that it was not part of the index LCIS [15]. Subsequent invasive breast cancers were classified to ipsilateral and contralateral invasive breast cancers. Subsequent invasive breast cancer was also subdivided by both ER and progesterone receptor (PR) status to examine the association between race/ethnicity and risk of invasive subtypes defined by both ER and PR.

### Statistical analysis

To compare categorical and continuous variables across racial/ethnic groups, we used the *χ*^2^ test and analysis of variance, respectively. Person-years were calculated from 6 months after the initial LCIS diagnosis until the diagnosis date of the second primary breast tumor (invasive or carcinoma in situ), death, or December 2015. The Kaplan-Meier estimates of 10-year probabilities of subsequent invasive breast cancer were computed for each of the four race/ethnic groups, and log-rank tests were performed to test for significant differences. We utilized the Lunn-McNeil competing risk models, an extension of the Cox proportional hazards regression models [21], to estimate the race-associated hazard ratio (HR) and 95% confidence interval (CI) of subsequent invasive breast cancer. Subsequent carcinoma in situ was modeled as separate competing outcomes. Specifically, each patient had a separate observation for each type of outcomes and the analysis was stratified on outcome types. Scaled Schoenfeld residuals were used to confirm the assumptions of proportionality in Cox models. The risks of ipsilateral and contralateral invasive breast cancer were analyzed using two separate competing risk models. We also analyzed subsequent invasive breast cancer subtypes defined by both ER and PR in black and white women using the aforementioned competing risk models; tumors positive for ER and/or PR were classified as hormone receptor-positive (ER+/PR+), and tumors negative for both ER and PR were classified as hormone receptor-negative (ER−PR−). Likelihood ratio tests for heterogeneity were used to determine statistically significant differences in the associations of race/ethnicity with cancer subtypes.

Each statistical model was adjusted for age (20–39, 40–49, 50–59, 60–69, or ≥ 70 years), year of LCIS diagnosis (1990–1999, 2000–2009, or 2010–2015), treatment of LCIS (no surgical treatment, breast-conserving surgery alone, breast-conserving surgery followed by radiation therapy, unilateral mastectomy, bilateral mastectomy, or unknown), and registries. The analyses were stratified by age at the diagnosis of LCIS (< 50 years vs ≥ 50 years). The interaction between race/ethnicity and age at the diagnosis of LCIS was assessed by including a cross-product term in multivariable-adjusted models. The statistical significance of an interaction term was evaluated by the likelihood ratio test. All statistical analyses were conducted with SAS (9.4 version). A two-sided *P* < 0.05 was used to indicate statistical significance.

## Results

Among 18,835 women with LCIS, 78.6% were white, 8.2% were black, 4.6% were Asian, and 8.6% were Hispanic. Mean age was 54.1 (a range of 20–95). They were followed for 90 months on average. While 9.9% of patients did not receive surgery, 83.0% had breast-conserving surgery alone, 1.4% were treated with breast-conserving surgery and radiation therapy, and 4.2% had a unilateral mastectomy.

Table 1 presents the characteristics of patients by race/ethnicity. Overall, women from racial/ethnic minority backgrounds were significantly younger at their LCIS diagnosis. Asian women were more likely than the other racial groups to undergo breast-conserving surgery alone, while white women were more likely than racial minority women to receive mastectomies.

**Table 1:** Characteristics of women with lobular carcinoma in situ (LCIS) in the SEER by race and ethnicity, 1990 to 2015 (*n* = 18,835)

	White	Black	Asian	Hispanic
Number of cases	14,811	1536	865	1623
Age at diagnosis, %				
Mean (SD)	54.7 (10.6)	53.3 (10.7)	51.0 (9.7)	51.5 (9.6)
20–39	3.3	5.7	6.2	5.3
40–49	32.9	34.8	45.4	44.4
50–59	35.3	34.1	31.7	31.0
60–69	17.7	16.8	11.2	13.7
≥ 70	10.8	8.6	5.4	5.6
Length of follow-up, %				
Median (range), months	95 (6–311)	80 (6–309)	74 (6–310)	77 (6–311)
6–11 months	4.2	5.5	6.1	6.2
12–59 months	27.3	32.7	35.1	33.8
60–119 months	30.2	30.4	30.5	30.6
≥ 120 months	38.3	31.5	28.2	29.4
Year of the first LCIS diagnosis, %				
1990–1999	19.2	16.3	12.5	13.8
2000–2009	53.2	47.5	46.8	48.1
2010–2015	27.7	36.3	40.7	38.1
Treatment				
No surgery	10.0	11.4	8.3	8.9
BCS alone	82.7	82.4	86.0	84.7
BCS and radiation	1.4	1.2	1.9	2.0
Mastectomy	4.5	3.5	2.8	3.2
Unknown	1.4	1.6	1.0	1.1

*Abbreviations*: *SD* standard deviation, *BCS* breast-conserving surgery, *ER* estrogen receptor, *PR* progesterone receptor

Race and ethnicity were classified into mutually exclusive categories of non-Hispanic white (hereafter referred to as white), non-Hispanic black (black), non-Hispanic Asian (Asian), and Hispanic (Hispanic)

**Fig. 1:**
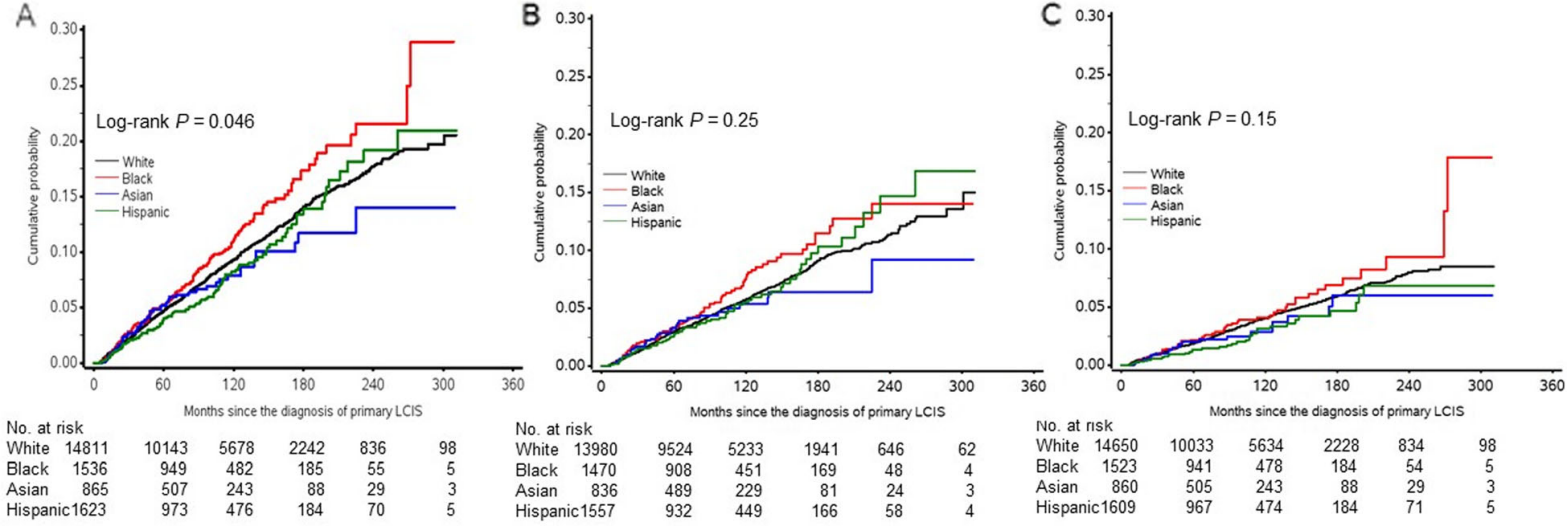
Cumulative incidences of subsequent invasive breast cancer in either breast or other body parts (**a**), ipsilateral invasive breast cancer (**b**), and contralateral invasive breast cancer (**c**) among four racial/ethnic groups of women with LCIS

Among 18,835 patients, 1567 (8.3%) subsequently developed invasive breast cancer in either breast (*n* = 1536) or other body parts (stage IV, *n* = 31) during the 90-month follow-up. Of these subsequent invasive tumors, 801 (51.1%) were ductal, 408 (26.0%) were lobular, and 242 (15.4%) had both lobular and other types of histology. We observed a significant racial/ethnic difference (*P* = 0.046) in the cumulative incidence of subsequent invasive breast cancer (Fig. 1a). The 10-year cumulative risk of subsequent invasive breast cancer was 7.9% in Asian women, 8.2% in Hispanic women, 9.3% in white women, and 11.2% in black women. The multivariable-adjusted RR of subsequent breast cancer was 1.33 (95% CI 1.11, 1.59) in black women compared to white women (Table 2). Asian and Hispanic women had a similar risk compared to white women. We also examined the race-associated risk in patients diagnosed and followed during three time intervals: between 1990 and 1999, between 2000 and 2009, and between 2010 and 2015 (Additional file 1: Table S1). The increased risk in black women was consistently observed over time. The analysis of race-associated risk of subsequent invasive breast cancer was also stratified by age at the diagnosis of LCIS; there was no significant variation in the association pattern between women diagnosed under 50 years and those diagnosed at the age of 50 years and older (Additional file 2: Table S2). Among the 17,843 women with breast-conserving surgery or no definitive surgery, 909 (5.1%) had ipsilateral invasive breast cancer during a median follow-up of 89 months (range 6-311 months). We observed no statistically significant difference in the cumulative incidence of ipsilateral invasive breast cancer by race/ethnicity (*P* = 0.20; Fig. 1b). Multivariable-adjusted analysis (Table 2) showed that black women had a significantly higher risk of ipsilateral invasive breast cancer when compared to white women (HR 1.37; 95% CI 1.08, 1.72). There was no significant difference in the risk of ipsilateral invasive breast cancer between Hispanic, Asian, and white women.

**Table 2:** Risk of subsequent invasive breast cancer overall and by laterality associated with race and ethnicity in women with LCIS

	Subsequent invasive breast cancer^a^			Ipsilateral invasive breast cancer^b^			Contralateral invasive breast cancer^c^		
	Person-years	Cases	HR^d^ (95% CI)	Person-years	Cases	HR^e^ (95% CI)	Person-years	Cases	HR^f^ (95% CI)
White	130,213	1264	1.00	120,334	722	1.00	128,949	521	1.00
Black	11,935	142	1.33 (1.11, 1.59)	11,283	85	1.37 (1.08, 1.72)	11,818	57	1.33 (1.00, 1.76)
Asian	6434	55	0.83 (0.62, 1.10)	6137	33	0.87 (0.61, 1.25)	6406	22	0.78 (0.49, 1.24)
Hispanic	12,229	106	0.89 (0.72, 1.09)	11,564	70	0.99 (0.77, 1.27)	12,142	36	0.76 (0.54, 1.08)

*Abbreviations*: *HR* hazard ratio, *95% CI* 95% confidence interval

^a^Subsequent invasive breast cancer included invasive breast cancer in either breast and metastatic breast cancer. The analysis included 19,545 women with LCIS

^b^The analysis was restricted to 17,843 women who did not have surgical treatment or received breast-conserving surgery for primary LCIS

^c^The analysis was restricted to 18,642 women who did not have surgical treatment or received breast-conserving surgery or unilateral mastectomy for primary LCIS

^d^HRs were adjusted for age (20–39, 40–49, 50–59, 60–69, or ≥ 70 years) and year of the initial LCIS diagnosis (1990–1999, 2000–2009, or 2010–2015), registry, and treatment for primary LCIS (no surgical treatment, breast-conserving surgery alone, breast-conserving surgery followed by radiation therapy, mastectomy, or unknown)

^e^The covariates were the same as the above. Treatment was categorized as no surgical treatment, breast-conserving surgery alone, or breast-conserving surgery followed by radiation therapy

^f^The covariates were the same as the above. Treatment was categorized as no surgical treatment, breast-conserving surgery alone, breast-conserving surgery followed by radiation therapy, or mastectomy

Among the 18,642 patients who had known treatment status, 635 (3.4%) patients were diagnosed with contralateral invasive breast cancer during a median follow-up of 91 months (range 6–311 months). There was no significant difference in the cumulative incidence of contralateral invasive breast cancer by race/ethnicity (*P* = 0.12; Fig. 1c). The multivariable-adjusted HR of contralateral invasive breast cancer in black women compared with white women was 1.33 (95% CI 1.00, 1.76), which was similar to the risk of subsequent invasive breast cancer and the risk of ipsilateral invasive breast cancer (Table 2). There was no significant difference in the risks of contralateral breast cancer in Asian women and Hispanic women compared with white women.

An analysis of hormone receptor status in subsequent invasive breast cancer was performed in white women and black women (*n* = 16,347) (Table 3). Black women had significantly higher risks of ER−PR− invasive breast cancer (HR 1.86; 95% CI 1.14, 3.03) and ER+/PR+ invasive breast cancer (HR 1.30, 95% CI 1.07, 1.59) following LCIS compared to white women. There was no significant variation in the associations with these two subtypes (*H*_eterogeneity_ = 0.15).

**Table 3:** Risk of subsequent hormone receptor-defined invasive breast cancer in black women with LCIS compared with white counterparts (*n* = 16,347)

	Person-years	ER+/PR+		ER− and PR−	
		Cases	HR^a^ (95% CI)	Cases	HR^a^ (95% CI)
White	130,213	1056	1.00	129	1.00
Black	11,935	115	1.30 (1.07, 1.59)	20	1.86 (1.14, 3.03)
				*H*_eterogeneity_ = 0.15	

*Abbreviations*: *HR* hazard ratio, *95% CI* 95% confidence interval, *ER* estrogen receptor, *PR* progesterone receptor

^a^HRs were adjusted for age (20–39, 40–49, 50–59, 60–69, or ≥ 70 years) and year of the primary LCIS diagnosis (1990–1999, 2000–2009, or 2010–2015), registry, and treatment for primary LCIS (no surgical treatment, breast-conserving surgery alone, breast-conserving surgery followed by radiation therapy, mastectomy, or unknown)

## Discussion

By utilizing SEER data from a racially diverse group of women with LCIS, we assessed the association between race/ethnicity and risks of subsequent invasive breast cancer among 19,545 patients. Compared with white women, black women had a significantly higher risk of developing invasive breast cancer during the follow-up. The risk of invasive breast cancer was increased by 37% in the ipsilateral breast and increased by 33% in the contralateral breast among black women. In addition, black women had significantly higher risks of developing ER+/PR+ and ER−PR− invasive breast cancer following LCIS. Asian and Hispanic women did not display a significant difference in the risk of acquiring subsequent invasive breast cancer when compared to white women.

We observed a 10-year incidence of subsequent invasive breast cancer that was 7.9% in Asian women, 8.2% in Hispanic women, 9.3% in white women, and 11.2% in black women. Other investigators have reported a similar cumulative long-term incidence of second breast tumors after LCIS, but ignored associations with race. A long-term follow-up study of 236 patients found that the probability of developing subsequent invasive carcinoma by 10 years after a diagnosis of LCIS was 13% [6]. Among 4853 women with LCIS identified from the 1973–1998 SEER database, there was a 7.1% minimum risk of developing subsequent invasive breast tumors on either the ipsilateral or contralateral breast [22]. Our findings of a higher 10-year incidence of subsequent invasive breast cancer among black women compared to other races support further investigation of the biological and non-biological factors that influence LCIS malignancy potential.

We assessed receptor expression in subsequent invasive breast cancer following LCIS between black women and white women, identifying that black women had a significantly higher risk for ER−PR− invasive subtypes. This correlated with other studies that have identified higher risks of these receptor statuses in black women [23]. The observation of higher risk of ER−/PR− invasive tumors following LCIS among black women is noteworthy due to the aggressiveness of those lesions. In a nationally representative population of DCIS patients diagnosed between 1990 and 2015, we demonstrated that compared with white women, the risk of developing ER−PR− invasive breast cancer was significantly increased by 86% (HR = 1.86, 95% CI 1.57–2.20) in black women and by 40% in Asian women (HR = 1.40, 95% CI 1.14–1.71). The associations for ER−/PR− invasive cancer were stronger than the associations for ER+/PR+ subtypes (HR = 1.31, 95% CI 1.21–1.43 in blacks; HR = 1.01, 95% CI 0.92–1.11 in Asians; *H*_eterogeneity_ = 0.0004) [24]. Thus, the magnitude of the associations between black race and risk of invasive breast cancer subtypes following LCIS and DCIS was similar. Among women with benign breast disease, African American identity was a significant risk factor for triple negative breast cancer [25]. These findings warrant further investigation of race-related biomarker profiling of pre-malignant breast lesions.

Within this study, differing treatment options were noted to be favored among patients of various races. Breast-conserving surgery alone was chosen by 83.0% of patients, 9.9% did not receive surgery, 4.2% had a unilateral mastectomy, and 1.4% were treated with both breast-conserving surgery and radiation therapy. Among the patients who chose mastectomies, white women were more likely to do so than women of racial minorities, and among the patients who chose no surgical interventions, black women were more likely to do so than women in the other racial groups. These racial variations in choices may be due to healthcare disparities, due to greater preferences of a certain type of treatment based on concomitant cultural norms, or due to the absence of a standardized treatment for LCIS based on the uncertainty regarding the lesion’s malignancy potential [26, 27]. The National Comprehensive Cancer Network (NCCN) guidelines (2018) states that surgical treatment is not required for classic LCIS but acknowledges clinician’s choice to perform surgical excision with negative margins for pleomorphic LCIS [26]. Considered as a more aggressive subtype, pleomorphic LCIS contains high-grade cytological features and usually is diagnosed by calcifications on mammography [28].

Overall, NCCN guidelines recommend counseling patients on lifestyle modifications and follow-up surveillance appointments that include annual mammography, physical examinations, and interval history every 6 to 12 months [26]. As a strategy of risk reduction, LCIS patients with higher risk, such as women with BRCA 1 or BRCA 2 genetic mutations, may undergo bilateral prophylactic mastectomy [10]. There is limited support for the addition of radiation therapy, but a small cohort study suggested some benefits [13]. Based on the more recent findings of Taylor et al. [29], there was no significant difference in overall survival between patients who received lumpectomy alone or lumpectomy with radiation treatment. Thus, considering the healthcare costs and potential health risk of radiation treatments, lumpectomy alone could be a more appropriate standardized treatment.

Adjuvant therapy such as chemoprevention is an effective method to reduce patient’s risk of developing breast cancer, specifically ER+ invasive breast cancer [30, 31]. The NSABP P-1 trial reported that tamoxifen reduced the incidence of invasive breast lesions from 42.5 per 1000 women in the placebo group to 24.8 per 1000 women in the treatment group [30]. The NCCN guidelines support the administration of tamoxifen, raloxifene, and aromatase inhibitors for 5 years as a risk reduction strategy in LCIS patients [26]. Chemoprevention has been associated with improved quality-adjusted life expectancy in premenopausal women when compared to postmenopausal women [32].

This study has limitations. SEER registries did not provide information regarding endocrine therapy utilization, family cancer history, comorbidities, obesity, alcohol consumption, breast cancer screening after LCIS, and socioeconomic status, which may influence the risk of second breast tumors. Although an adequate analysis was conducted from the LCIS patient data, receptor status in LCIS lesions, tumor grade, and tumor size information were unavailable for more than half of patients and thus were not accounted for in the analysis. Treatment status was missing in less than 2% of patients. Missing indicators were documented for this analysis. This approach has been demonstrated to have no significant impacts on the estimated associations between exposures and cancer outcomes when missing is less than 50% [33].

## Conclusions

Our study provides the largest population-based analysis on the association between race/ethnicity and the development of subsequent invasive breast cancer after LCIS and for the first time addresses the risk of hormone receptor-negative breast cancer following LCIS. Black women with LCIS had an elevated risk of subsequent invasive breast cancer, including both ER+/PR+ and ER−PR− tumors. Asian and Hispanic women did not have a higher risk of developing these lesions when compared to white women. Further research may refine post-treatment surveillance strategies and might better understand racial preferences for and provider-patient communications about LCIS treatment. In addition, studies to identify which gene expression and molecular alterations in LCIS are associated with the risk of developing invasive carcinoma may open new pathways for chemoprevention and improve patient care to reduce the disproportionate burden of breast cancer in black women. These results also highlight the importance of understanding genetic background, early-life environmental/behavioral exposures, and their interactions as contributors to racial differences in risk of developing invasive breast cancer following LCIS.

## Supplementary information

**Additional file 1: Table S1.** The race-associated hazards ratios of subsequently developing invasive breast cancer in women with LCIS during three time intervals.

**Additional file 2: Table S2.** The race-associated hazard ratios of subsequent invasive breast cancer in women with LCIS by age at the diagnosis of LCIS.

## Data Availability

The dataset analyzed during the current study are available in the NCI’s SEER https://seer.cancer.gov/data/access.html.

## Abbreviations

CI: Confidence interval
DCIS: Ductal carcinoma in situ
ER: Estrogen receptor
HR: Hazard ratio
LCIS: Lobular carcinoma in situ
PR: Progesterone receptor
SEER: Surveillance, Epidemiology, and End Results
