# Supplementary material for: Associations of race and ethnicity with risk of developing invasive breast cancer after lobular carcinoma in situ
Source: Breast Cancer Res. 2019 Nov 14;21:120. doi: 10.1186/s13058-019-1219-8 (PMC6854630; doi:10.1186/s13058-019-1219-8)
Supplement: Supplementary file 1 — Additional file 1: Table S1. The race-associated hazards ratios of subsequently developing invasive breast cancer in women with LCIS during three time intervals. [file 13058_2019_1219_MOESM1_ESM.docx]

Additional file 1

The race-associated hazards ratios of subsequently developing invasive breast cancer in women with LCIS during three time intervals.

| Calendar years at diagnosis and followup | No. of cases | Black | | Asian | | Hispanic | |
| --- | --- | --- | --- | --- | --- | --- | --- |
|  |  | HR^a^ | 95% CI | HR^a^ | 95% CI | HR^a^ | 95% CI |
| 1990-1999 | 3420 | 1.21 | 0.88, 1.67 | 0.62 | 0.35, 1.11 | 1.02 | 0.72, 1.44 |
| 2000-2009 | 9793 | 1.35 | 1.06, 1.71 | 0.82 | 0.56, 1.21 | 0.78 | 0.58, 1.03 |
| 2010-2015 | 5622 | 1.40 | 0.85, 2.30 | 1.25 | 0.67, 2.35 | 1.09 | 0.63, 1.88 |

Abbreviations: HR, hazards ratio; CI, confidence interval.

^a^ HRs were adjusted for age at the diagnosis of initial LCIS (20-39, 40-49, 50-59, 60-69, or ≥70 years), registries, and treatment for primary LCIS (no surgical treatment, breast-conserving surgery alone, breast-conserving surgery followed by radiation therapy, mastectomy, or unknown).
