# Supplementary material for: Associations of race and ethnicity with risk of developing invasive breast cancer after lobular carcinoma in situ
Source: Breast Cancer Res. 2019 Nov 14;21:120. doi: 10.1186/s13058-019-1219-8 (PMC6854630; doi:10.1186/s13058-019-1219-8)
Supplement: Supplementary file 2 — Additional file 2: Table S2. The race-associated hazard ratios of subsequent invasive breast cancer in women with LCIS by age at the diagnosis of LCIS. [file 13058_2019_1219_MOESM2_ESM.docx]

Additional file 2

The race-associated hazards ratios of subsequently developing invasive breast cancer in women with LCIS by age at the diagnosis of LCIS.

| Age at the LCIS diagnosis | No. of cases | Black | | Asian | | Hispanic | |
| --- | --- | --- | --- | --- | --- | --- | --- |
|  |  | HR^a^ | 95% CI | HR^a^ | 95% CI | HR^a^ | 95% CI |
| <50 years | 7237 | 1.19 | 0.89, 1.58 | 0.64 | 0.40, 1.00 | 0.74 | 0.54, 1.01 |
| ≥50 years | 11598 | 1.45 | 1.16, 1.82 | 1.01 | 0.70, 1.46 | 1.01 | 0.78, 1.33 |
|  |  | Pinteraction=0.08 | | | | | |

Abbreviations: HR, hazards ratio; CI, confidence interval.

^a^ HRs were adjusted for age at the diagnosis of initial LCIS (20-39, 40-49, 50-59, 60-69, or ≥70 years), registries, and treatment for primary LCIS (no surgical treatment, breast-conserving surgery alone, breast-conserving surgery followed by radiation therapy, mastectomy, or unknown)
